# Increased Risk of Chronic Kidney Disease Associated With Weight Gain in Healthy Adults: Insight From Metabolic Profiles and Body Composition

**DOI:** 10.3389/fmed.2021.705881

**Published:** 2021-09-28

**Authors:** Hae-Ryong Yun, Hyung Woo Kim, Tae Ik Chang, Ea Wha Kang, Young Su Joo, Ki Heon Nam, Hyoungnae Kim, Jung Tak Park, Tae-Hyun Yoo, Shin-Wook Kang, Seung Hyeok Han

**Affiliations:** ^1^Division of Nephrology, Department of Internal Medicine, Yongin Severance Hospital, Yonsei University College of Medicine, Seoul, South Korea; ^2^Department of Internal Medicine, College of Medicine, Institute of Kidney Disease Research, Yonsei University, Seoul, South Korea; ^3^Department of Internal Medicine, National Health Insurance Service Medical Center, Ilsan Hospital, Goyang, South Korea; ^4^Division of Nephrology, Soonchunhyang University Hospital, Seoul, South Korea; ^5^Department of Internal Medicine, College of Medicine, Severance Biomedical Science Institute, Brain Korea 21 PLUS, Yonsei University, Seoul, South Korea

**Keywords:** body mass index, group-based trajectory modeling, chronic kidney disease, metabolic profiles, body composition

## Abstract

**Objective:** Obesity is an established risk factor for kidney damage. In this study, we explored the long-term association of changes in body mass index (BMI) over time with incident chronic kidney disease (CKD).

**Methods:** For this analysis, 5,393 middle-aged adults without comorbidities in the Korean Genome and Epidemiology Study (KoGES) were included. Group-based trajectory modeling was used to determine the patterns of BMI change (decreasing, stable, and increasing BMI) between baseline and year 4. The primary outcome was the subsequent development of CKD from year 4. A multivariable Cox proportional hazards model was constructed to determine the risk of incident CKD according to BMI trajectories.

**Results:** During 55,327 person-years, incident CKD occurred in 354 (6.5%) participants; 6.0, 6.1, and 7.8 per 1,000 person-years across the trajectories, respectively (*P* = 0.005). In the multivariable-adjusted Cox proportional hazards model, the increasing BMI trajectory was associated with a 1.4-fold [hazard ratio (HR), 1.41; 95% CI, 1.06–1.87] a higher risk of incident CKD compared with stable BMI trajectory. This association was stronger for overweight and obese individuals. The HRs for CKD development in these two groups were 1.6 (95% CI, 1.06–1.87) and 2.2 (95% CI, 1.40–3.48), respectively. While the increasing BMI group was gaining weight, there were concomitant increases in blood pressure, insulin resistance, serum concentrations of total cholesterol, triglyceride, and high-sensitivity C-reactive protein (hs-CRP), and fat mass, but high-density lipoprotein (HDL)-cholesterol level and muscle-to-fat (MF) ratio decreased.

**Conclusion:** Weight gain is associated with an increased risk of incident CKD in healthy adults. This association is attributed to worsening metabolic profiles and increasing fat mass.

## Introduction

Over the past 5 decades, obesity has become a serious health concern (1). The estimated direct and indirect costs associated with obesity among adults account for 20.6% of the United States healthcare expenditure or US$209 billion (2). More importantly, the prevalence of obesity is expected to increase by 40% worldwide in the next decade (3). Obese individuals are highly likely to have metabolic disturbances, including hypertension, diabetes, dyslipidemia, and inflammation, and obesity itself constitutes a major component of metabolic syndrome (4–7). In addition, subjects with metabolic syndrome have a 2.6-fold higher risk of incident chronic kidney disease (CKD) than healthy individuals (8). Interestingly, the prevalence of these comorbid conditions has increased concomitantly with obesity (9). A high body mass index (BMI) is a major risk factor for adverse renal outcomes, including a decreased estimated glomerular filtration rate (eGFR), development of CKD, and progression to end-stage kidney disease (10–12). Obesity-induced glomerular hyperfiltration, hypertension, and metabolic disturbances underlie the negative effects of obesity on the kidney, all of which can cause filtration barrier injuries and eventually, proteinuria (13–16).

Major shortcomings of studies regarding obesity include the use of cross-sectional analysis, simple definition for the changes in body weight during fixed time frames, and failure to account for dynamic changes in weight over various time periods. In addition, the association of weight changes with the new development of CKD has seldom been tested in healthy adults. Thus, we aimed to explore the pure association between changes in weight and incident CKD among middle-aged Korean individuals without medical disease according to three different trends of BMI over 11 years using the database from the large-scale prospective cohort study. We further evaluated the association between trends in BMI and changes in metabolic profiles and body composition.

## Materials and Methods

### Study Design and Population

The Korean Genome and Epidemiology Study (KoGES) is a consortium project launched in 2001 by Korea's National Research Institute of Health, Centers for Disease Control and Prevention, and the Ministry of Health and Welfare, and includes six prospective cohort studies categorized into population-based and gene-environment model studies. The rationale, design, methods, and protocol summary of the KoGES are described in detail elsewhere (17). Briefly, 10,030 Korean adults aged between 40 and 69 years, comprising 5,012 (49.9%) residents from Ansan (urban area) and 5,018 (50.1%) residents from Ansung (rural area) were enrolled between 2001 and 2002. All participants provided informed consent. They underwent government-sponsored medical health check-ups and undertook different surveys at the baseline. Serial follow-up assessments of the biochemical data of the participants were performed biennially from 2001 to 2014. KoGES was conducted in accordance with the Declaration of Helsinki and approved by the Institutional Review Board of Yonsei University Health System's Clinical Trial Center (approval number: 4-2016-0100).

The participants who had attended <2 clinic visits (*n* = 965) were excluded. We also excluded 2,797 participants with BMI <18.5 kg/m^2^ (*n* = 246), prior kidney disease or baseline eGFRs of <60 ml/min/1.73 m^2^ (*n* = 342), prior hypertension or use of blood pressure-lowering agents (*n* = 1,304), history of diabetes or use of blood glucose-lowering agents (*n* = 447), history of dyslipidemia or use of lipid-lowering agents (*n* = 226), prior cardiovascular events (*n* = 123), or albuminuria (*n* = 109). We also excluded individuals with missing variables, development of renal outcome within the third visit, and loss of follow-up within 4 years (*n* = 875). Consequently, 5,393 healthy participants who did not have a medical disease at the baseline were included in the present study (Supplementary Figure 1).

### Data Collection

A standardized self-administered questionnaire was used to collect demographic data of each participant (age, sex, medical history, medication use, alcohol consumption habit, smoking habit, level of education, and monthly income). Smoking and alcohol consumption habits were divided into two categories as never or ever (including current or former). The education level was classified into three categories: primary, middle or high secondary, and post-secondary. Income per month was divided into four categories; < $850 per month; $850 to $1,700 per month; and >$1,700 per month. Trained nurses measured the BP levels using a standard mercury sphygmomanometer while the participants were seated. Height was measured to the nearest 0.1 cm with a stadiometer, while the participants stood barefoot. Weight was measured while the participants wore light clothes and no shoes. The BMI was calculated by dividing the initial weight (kg) by height squared (m^2^), and it was categorized as normal weight (BMI 18.5–22.9 kg/m2), overweight (BMI 23–24.9 kg/m^2^), or obese (BMI ≥ 25 kg/m^2^) using the International Association for the Study of Obesity, International Obesity Task Force (2000), and Committee of Clinical Practice Guidelines and Korean Society for the Study of Obesity (18, 19). Blood and urine samples obtained after an 8 h fast were transported to Seoul Clinical Laboratories, Seoul, Republic of Korea, within 24 h of sampling to minimize laboratory errors, and the hemoglobin, blood urea nitrogen, creatinine, total cholesterol, triglyceride, and high-density lipoprotein-cholesterol (HDL-C) were measured using Hitachi 747 chemistry analyzer (Hitachi Ltd, Tokyo, Japan). High-sensitivity C-reactive protein (hs-CRP) levels were measured by immunoradiometric assay (ADVIA 1650, Bayer Diagnostics, Tarrytown, NY, USA). The low-density lipoprotein cholesterol (LDL-C) levels were calculated using the following formula: [total cholesterol (mg/dl)—HDL-C (mg/dl)—triglyceride (mg/dl)/5]. The serum insulin concentrations were measured by radioimmunoassay (LINCO kit, St Charles, MO, USA). Insulin resistance was assessed with the homeostasis model assessment of insulin resistance (HOMA-IR) equation [fasting insulin (μIU/ml) × fasting glucose (mg/dl)/405] (20). A dipstick urine test was used for the detection of albuminuria or microscopic hematuria. The serum creatinine level was measured using the Jaffe method throughout the study period. Thus, we converted non-isotope dilution-mass spectrometry (IDMS) creatinine to IDMS creatinine (21, 22). Then, eGFRs were calculated using the CKD-Epidemiology Collaboration equation (22). The laboratory parameters were measured at the baseline and every 2 years thereafter. Trained staff performed all the measurements, including the anthropometric parameter measurements.

### Assessment of Body Composition

Body composition was assessed using multi-frequency bioelectrical impedance analysis (BIA; InBody 3.0, Biospace, Seoul, Korea). Detailed information for BIA is described elsewhere (23). The muscle mass was expressed as the muscle mass index (MMI, muscle mass/height^2^). The fat mass was expressed as the fat mass index (FMI, fat mass/height^2^). The muscle-to-fat (MF) ratio was defined as MMI/FMI.

### Exposure and the Primary Endpoint

**Figure 1:**
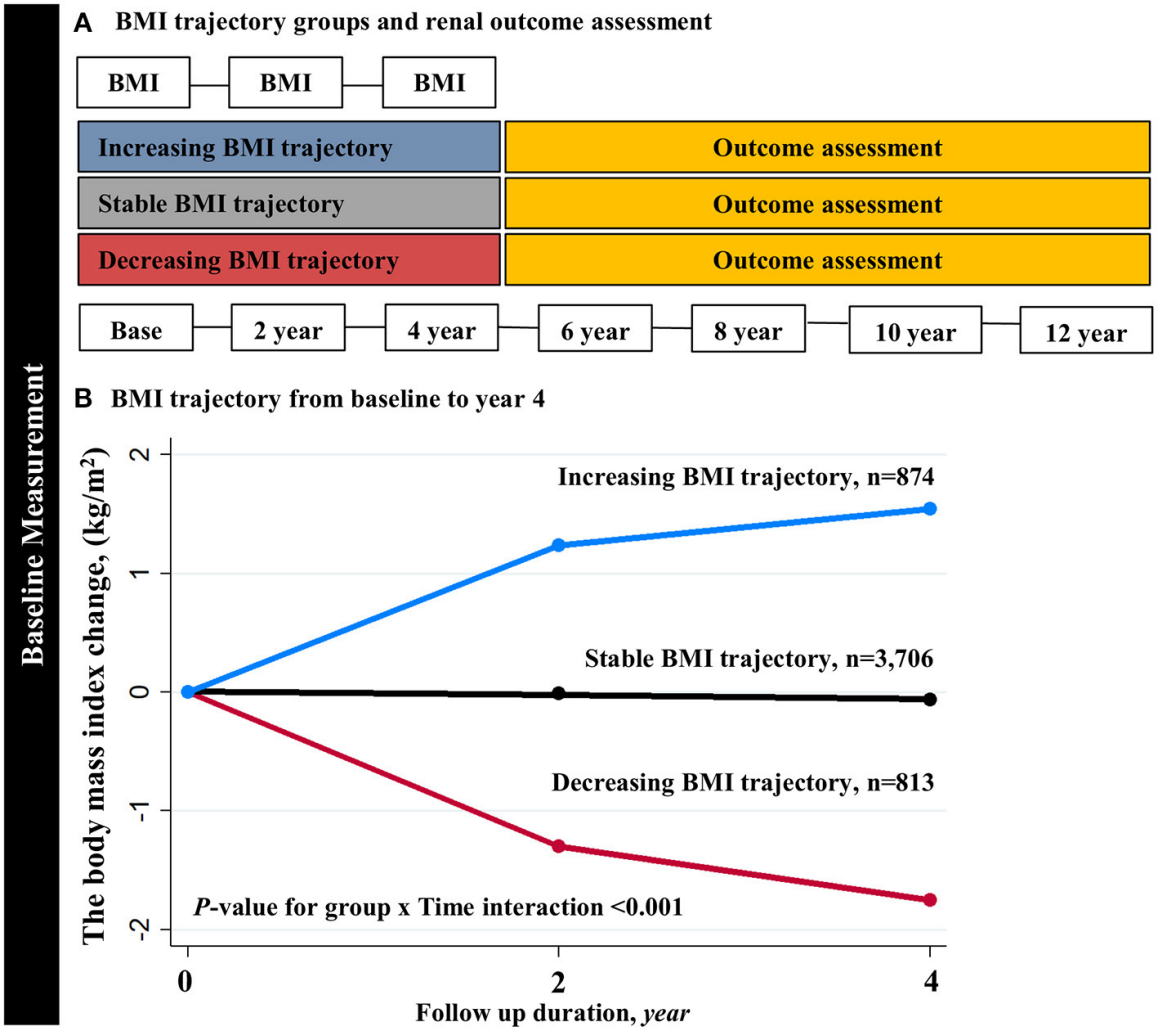
Trajectory modeling identified three distinct BMI trajectory groups. **(A)** The primary analysis derived BMI trajectory groups using the first 4-year BMI measurements. **(B)** The slopes of BMI trajectories were significantly different across the three groups (*P-*value for group × Time interaction <0.001). BMI, body mass index.

This study's main exposure is changes in BMI over time. The primary outcome was incident CKD, which was defined as two consecutive eGFRs <60 ml/min/1.73 m^2^ that occurred during the follow-up period, the first of which was designated as the endpoint. To evaluate the association of early BMI changes with the subsequent CKD development that occurred at a later time point, we analyzed only the adverse kidney events that occurred after 4 years of the BMI trajectory assessment period (Figure 1A).

### Trajectory Analysis

To determine BMI trajectories, we used group-based trajectory modeling (GBTM) (24–26). It assumes that the participants are composed of multiple groups having trajectories that follow the same development course on the outcome of interest. According to these assumptions, time-dependent covariates explain variation in the average trajectory within each group. In this study, we used three BMI measurements at the baseline, and years 2 and 4. The heterogeneity of the slopes of BMI trajectory groups was tested using the “TRAJTEST” macro. Model fit was assessed with the following methods: (1) the Bayesian Information Criterion (BIC), (2) the number of participants in each trajectory (>5% overall population), and (3) average probability of final group membership across the trajectory groups to assess the discrimination power of the individual model. We constructed models with different numbers of trajectory groups and different forms of potential trajectories (linear, quadratic, or cubic). We started a model with 3, 4, and 5 trajectories and then evaluated different functional forms to the enhanced model fit and calculated the posterior probabilities for each number of participants in several trajectory groups to test the discrimination power of the model (Supplementary Table 1). When the number of trajectories was ≥4, the BIC was decreased, and the number of participants in each trajectory was also decreased, resulting in less discrimination power across the trajectory groups. Finally, we identified three BMI trajectory groups according to the visual appearance and clinically meaningful BMI trend: decreasing BMI (slope of BMI per year (%) = −0.47, 95% CI −0.46 to −0.48; *p* < 0.001), stable BMI (slope = 0.001, 95% CI 0.001–0.002; *P* = 0.014), and increasing BMI (slope = 0.50, 95% CI 0.49–0.50; *p* < 0.001) (Figure 1B). The BMI slopes were significantly different across the three groups (*P*-value for group × time interaction <0.001).

### Statistical Analysis

The continuous variables are expressed as the means and SDs or as the medians with interquartile ranges (IQRs). The categorical variables are expressed as numbers and percentages. The baseline characteristics of the participants who were grouped according to different trajectories were compared using an ANOVA for continuous variables and the chi-square test for categorical variables. Person-years of follow-up were calculated from the baseline until the date of incident CKD or the last visit. The Kaplan-Meier analysis and log-rank test were used to estimate the cumulative renal survival rates. Survival time was defined as the time interval between enrollment and the onset of outcome events. Multivariable Cox proportional hazards models were constructed to assess the associations between the trajectories of BMI and incident CKD with three adjustment levels. Violation of the proportional hazards assumption was tested by inspection of log [–log (survival)] curves. Model 1 represented the crude hazard ratios (HRs) without adjustments. Model 2 included baseline age and sex. Model 3 incorporated the area of residence, smoking habits, alcohol consumption habits, income, education level, BMI, mean arterial pressure (MAP), hemoglobin, glucose, and eGFR. Model 4 was constructed after further adjustments to include HDL-C, triglyceride, and hs-CRP levels. The results from multivariable Cox models are presented as the HRs and 95% CIs.

In sensitivity analysis, we examined the association of three 4 year BMI trajectories with different outcome assessment periods (4–8 years). We further tested the association between changes in BMI and incident CKD among prespecified subgroups by sex (male or female), age (<50 or ≥50 years), smoking status, alcohol consumption habit, and baseline BMI (18.5–22.9, 23.-24.9, or ≥25. kg/m^2^). We also performed additional sensitivity analysis after excluding 534 subjects with isolated microscopic hematuria. Restricted cubic splines were used to reveal the association between percent BMI changes as continuous variables and risk of incident CKD. The rate of eGFR decline per year was assessed using the slope of eGFR obtained from a generalized linear mixed model. The statistical analyses were performed using Stata, version 15.1 (Stata Corporation, College Station, TX). A *p* < 0.05 was considered statistically significant.

## Results

### Baseline Characteristics Among Three Trajectories of BMI

Table 1 presents the baseline characteristics of the participants according to the trajectories of changes in BMI. During the BMI assessment period, BMI decreased to −7.5% from the baseline in the decreasing BMI group, whereas it increased to 7.6% from the baseline in the increasing BMI group. The difference in BMI and the percent changes of BMI remained constant after the BMI assessment period (Supplementary Table 2). The 5,393 participants included 2,636 (48.9%) men, and the mean (SD) age was 50.8 (8.5) years. The baseline BMI was significantly lower in the increasing BMI group than in the stable and decreasing groups. The increasing BMI group had a lower proportion of obese participants and lower levels of baseline BP, fasting glucose, insulin resistance, total cholesterol, triglyceride, and hs-CRP levels. On the contrary, the baseline eGFR and HDL-cholesterol levels were significantly higher in the increasing BMI group than in the decreasing group. MMI and FMI were lower in the increasing BMI group. However, the MF ratio was significantly higher in the increasing BMI group compared with the stable and decreasing BMI group.

**Table 1:** Baseline characteristics of the trajectories of changes in the body mass index (BMI) groups.

		**Trajectories of changes in BMI groups**			
	**Overall**	**Decreasing BMI**	**Stable BMI**	**Increasing BMI**	***P*-value**
Participants, No. (%)	5,393	874	3,706	813	
Age, mean (SD), years	50.8 (8.5)	50.3 (8.28)	50.9 (8.5)	51.1 (8.6)	0.11
Sex, No. (%)					<0.001
Men	2,636 (48.9)	377 (43.1)	1,894 (51.1)	365 (44.9)	
Women	2,757 (51.1)	497 (56.9)	1,812 (48.9)	448 (55.1)	
Residence, No. (%)					<0.001
Ansung	2,715 (50.3)	342 (39.1)	1,863 (50.3)	510 (62.7)	
Ansan	2,678 (49.7)	532 (60.9)	1,843 (49.7)	303 (37.3)	
Smoking habit, No. (%)					<0.001
Never	3,155 (58.5)	552 (63.2)	2,130 (57.5)	473 (58.2)	
Ever	2,238 (41.5)	322 (36.8)	1,576 (42.5)	340 (41.8)	
Alcohol consumption habit, No. (%)					0.10
Never	2,408 (44.7)	405 (46.3)	1,612 (43.5)	391 (48.1)	
Ever	2,985 (55.4)	469 (53.7)	2,094 (56.5)	422 (51.9)	
Education level, No. (%)					<0.001
Primary	1,593 (29.8)	231 (26.6)	1,069 (29.1)	293 (36.1)	
Middle or High secondary	3,030 (56.6)	508 (58.6)	2,067 (56.2)	455 (56.1)	
Post-secondary	731 (13.7)	128 (14.8)	540 (14.7)	63 (7.8)	
Household income per month, No. (%)					<0.001
<850 $	3,292 (62.2)	708 (61.4)	2,205 (60.6)	559 (70.3)	
850–1,700 $	1,585 (30.0)	264 (30.7)	1,122 (30.9)	199 (25.0)	
>1,700 $	415 (7.8)	68 (7.9)	309 (8.5)	38 (4.8)	
Follow-up duration, mean (SD), years	10.2 (2.6)	10.1 (2.6)	10.3 (2.5)	10.1 (2.8)	0.04
Baseline BMI, mean (SD), kg/m^2^	24.3 (3.1)	25.8 (3.2)	24.1 (2.9)	23.5 (3.1)	<0.001
Changes in BMI^a^, mean (SD), %	−0.3 (5.4)	−7.5 (3.4)	−0.1 (2.7)	7.6 (3.9)	<0.001
Baseline BMI category, No. (%)					<0.001
Normal weight (18.5–22.9 kg/m^2^)	1,829 (34.0)	159 (18.2)	1,301 (35.1)	369 (45.3)	
Overweight (23.0–24.9 kg/m^2^)	1,467 (27.2)	186 (21.3)	1,075 (29.0)	206 (25.3)	
Obese (≥25.0 kg/m^2^)	2,097 (38.9)	529 (60.5)	1,330 (35.9)	238 (29.3)	
Blood pressures, mean (SD)					
Systolic, mmHg	118.1 (16.3)	121.0 (17.6)	117.6 (16.0)	117.1 (15.9)	<0.001
Diastolic, mmHg	78.8 (10.8)	80.8 (11.1)	78.6 (10.7)	77.5 (10.4)	<0.001
MAP, mmHg	91.3 (12.0)	94.3 (12.6)	91.6 (11.9)	90.7 (11.6)	<0.001
Laboratory results, mean (SD)					
Hemoglobin, g/dL	13.5 (1.5)	13.5 (1.6)	13.6 (1.5)	13.4 (1.5)	0.01
Blood urea nitrogen, mg/dL	14.1 (3.5)	14.0 (3.4)	14.1 (3.5)	14.2 (3.5)	0.72
Creatinine, mg/dL	0.8 (0.1)	0.8 (0.1)	0.8 (0.1)	0.8 (0.1)	0.90
eGFR, mL/min per 1.73 m^2^	93.9 (12.7)	93.2 (12.9)	93.8 (12.7)	95.1 (12.2)	0.007
Fasting glucose, mg/dL	89.4 (15.6)	91.7 (16.8)	89.1 (14.8)	88.7 (17.1)	<0.001
HbA1c, %	5.6 (0.5)	5.6 (0.6)	5.5 (0.5)	5.6 (0.6)	<0.001
HOMA-IR^b^	1.6 (1.1)	1.8 (1.1)	1.6 (1.2)	1.5 (0.8)	<0.001
Albumin, g/dL	4.2 (0.3)	4.2 (0.3)	4.2 (0.3)	4.1 (0.2)	<0.001
hs-CRP^c^, median (IQR), mg/L	1.3 (0.6–2.3)	1.5 (0.7–2.5)	1.3 (0.6–2.3)	1.2 (0.5–2.1)	<0.001
Lipid profiles, mean (SD)					
Total cholesterol, mg/dL	188.6 (34.0)	193.8 (35.4)	188.6 (33.6)	182.8 (33.8)	<0.001
Triglyceride, mg/dL	153.6 (98.1)	165.7 (106.1)	153.4 (99.9)	141.8 (77.0)	<0.001
HDL-cholesterol, mg/dL	45.1 (9.9)	44.2 (9.5)	45.2 (9.9)	46.1 (10.1)	<0.001
LDL-cholesterol, mg/dL	116.9 (33.1)	120.8 (33.9)	116.9 (32.8)	112.5 (32.6)	<0.001
Body composition					
MMI (muscle mass/height^2^)	43.8 (8.0)	44.4 (7.9)	43.8 (8.0)	42.4 (7.6)	<0.001
FMI (fat mass/height^2^)	16.5 (5.3)	19.2 (5.6)	17.2 (4.9)	15.0 (5.3)	<0.001
MF ratio (MMI/FMI)	2.6 (1.1)	2.3 (1.0)	2.5 (1.1)	3.0 (1.3)	<0.001

*Variables for categorical variables are given as the number (percentage) of participants unless otherwise indicated*.

*To convert serum creatinine to micromoles per liter, multiply by 88.4; to convert to urea nitrogen to millimoles per liter, multiply by 0.357; to convert glucose to millimoles per liter, multiply by 0.0555; to convert total, HDL, and LDL cholesterol to millimoles per liter, multiply by 0.0259; and to convert triglycerides to millimoles per liter, multiply by 0.0113*.

a *Changes in BMI were calculated as the percent BMI changes between the baseline and the third visit*.

b *HOMA-IR was calculated according to the formula; fasting insulin (μIU/ml) × fasting glucose (mg/dL)/405*.

c *Variables were compared by the Kruskal-Wallis method*.

*BMI, body mass index; MAP, mean arterial pressure; eGFR, estimated glomerular filtration rate; HOMA-IR, homeostasis model assessment for insulin resistance; hs-CRP, high-sensitivity C-reactive protein; HDL, high-density lipoprotein; LDL, low-density lipoprotein; MMI, muscle mass index; FMI, fat mass index; MF, muscle-to-fat*.

### Risk of Incident Chronic Kidney Disease

**Figure 2:**
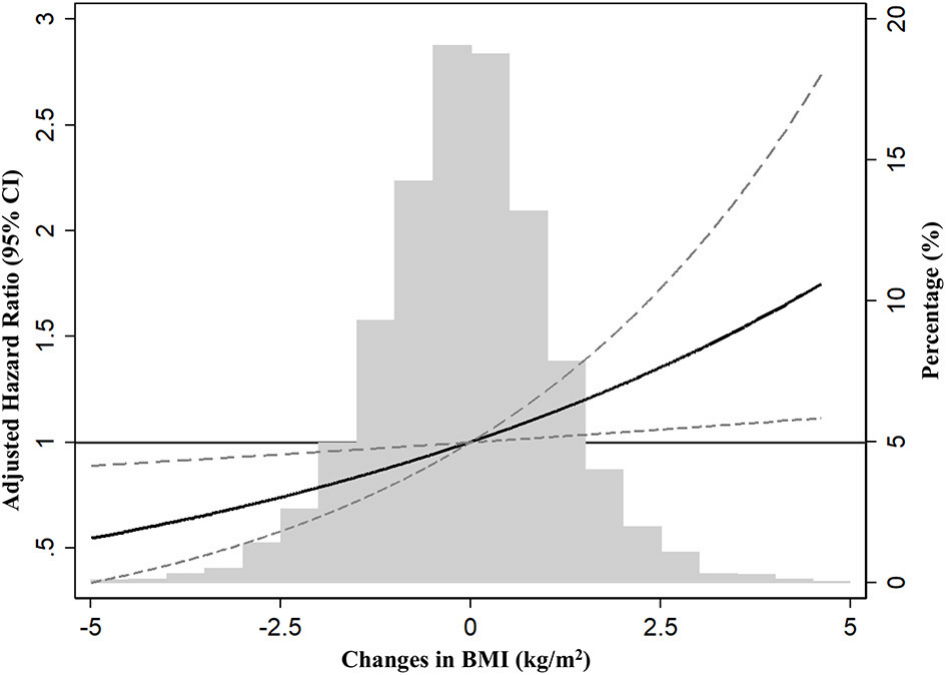
Restrict cubic spline analysis for risk of incident chronic kidney disease (CKD) by changes in BMI (%). HR was expressed after adjustment of covariates, including age, sex, the area of residence, smoking, alcohol consumption habit, income, education levels, body mass index, mean arterial pressure, hemoglobin, albumin, fasting glucose, estimated glomerular filtration rate, high-density lipoprotein-cholesterol, triglyceride, and high-sensitive C-reactive protein. BMI, body mass index; CKD, chronic kidney disease; CI, confidence interval.

During 55,327 person-years (median 11.5 years), incident CKD occurred in 354 (6.5%) participants, and the overall CKD incidence rate was 6.3 per 1,000 person-years. Among three trajectory groups, the incidence rates of CKD were 6, 6.1, and 7.8 per 1,000 person-years in decreasing, stable, and increasing trajectory groups, respectively (*P* = 0.005). The unadjusted HRs did not differ among groups (Model 1). However, in the multivariable Cox proportional hazard model after sequential adjustment of demographics and repeated laboratory measures, the HR for incident CKD was 1.39 (95% CI 1.04–1.86) in the increasing BMI group compared with the stable group (Model 3). In the model, further adjusted for metabolic parameters such as HDL-C, triglyceride, and hs-CRP, the increasing BMI group had a 1.45-fold (HR, 1.45; 95% CI, 1.08–1.94) higher risk of CKD development compared with the stable group (Table 2). In addition, the decreasing BMI group had a 39% (HR,0.61; 95% CI,0.41–0.89; *P* = 0.01) lower risk of CKD development when compared with the increasing BMI group (Supplementary Table 3). In the adjusted Kaplan-Meier plot, the cumulative incidence of CKD development was significantly higher in the increasing BMI group (Supplementary Figure 2). In sensitivity analysis with an outcome assessment period of years 4–8, the HR was 1.63 (95% CI, 1.15–2.31) in the increasing BMI group compared with the stable group (Supplementary Table 4). In additional sensitivity analysis after excluding 534 subjects with isolated microscopic hematuria, increasing BMI trajectory also showed a higher risk of incident CKD than stable trajectory (HR 1.39, 95% CI, 1.03–1.91) (Supplementary Table 5). The restricted cubic spline curve after full adjustment of confounding factors also showed a graded increase in HRs in proportion to changes in BMI (Figure 2). Finally, the slope of eGFR decline per year [ml/min/1.73 m^2^ (95% CI)] was significantly greater in the increasing BMI trajectory [−1.28 (−1.42 to −1.15)] compared with stable trajectory [−1.01 (−1.09 to −0.97)] (Supplementary Table 6).

**Table 2:** Hazard ratios for the incident chronic kidney disease according to the body mass index trajectory groups.

	**Events**	**Person-years**	**Incidence rate 1,000 person-years**	**Delta BMI (%)**	**Model 1**	**Model 2**	**Model 3**	**Model 4**
Decreasing	54	8,909	6.0	−5.6 (−8.8 to−2.0)	0.98 (0.72–1.31)	1.0 (0.77–1.40)	0.91 (0.67–1.24)	0.89 (0.65–1.20)
Stable	236	38,227	6.1	−0.2 (−0.5 to 0.2)	Reference	Reference	Reference	Reference
Increasing	64	8,191	7.8	5.6 (1.9 to 9.3)	1.27 (0.96–1.68)	1.30 (0.99–1.78)	1.39 (1.04–1.86)	1.45 (1.08–1.94)

*Model 1: a crude analysis without adjustment*.

*Model 2: an adjusted model including age and sex*.

*Model 3: Model 2 plus the area of residence, smoking habit, alcohol consumption habits, income, education level, body mass index, mean arterial pressure, hemoglobin, fasting glucose, and estimated glomerular filtration rate*.

*Model 4: Model 2 plus high-density lipoprotein-cholesterol, triglyceride, and high-sensitive C-reactive protein*.

*CKD, chronic kidney disease; BMI, body mass index; HRs, hazard ratios; CI, confidence interval*.

### Subgroup Analysis

**Figure 3:**
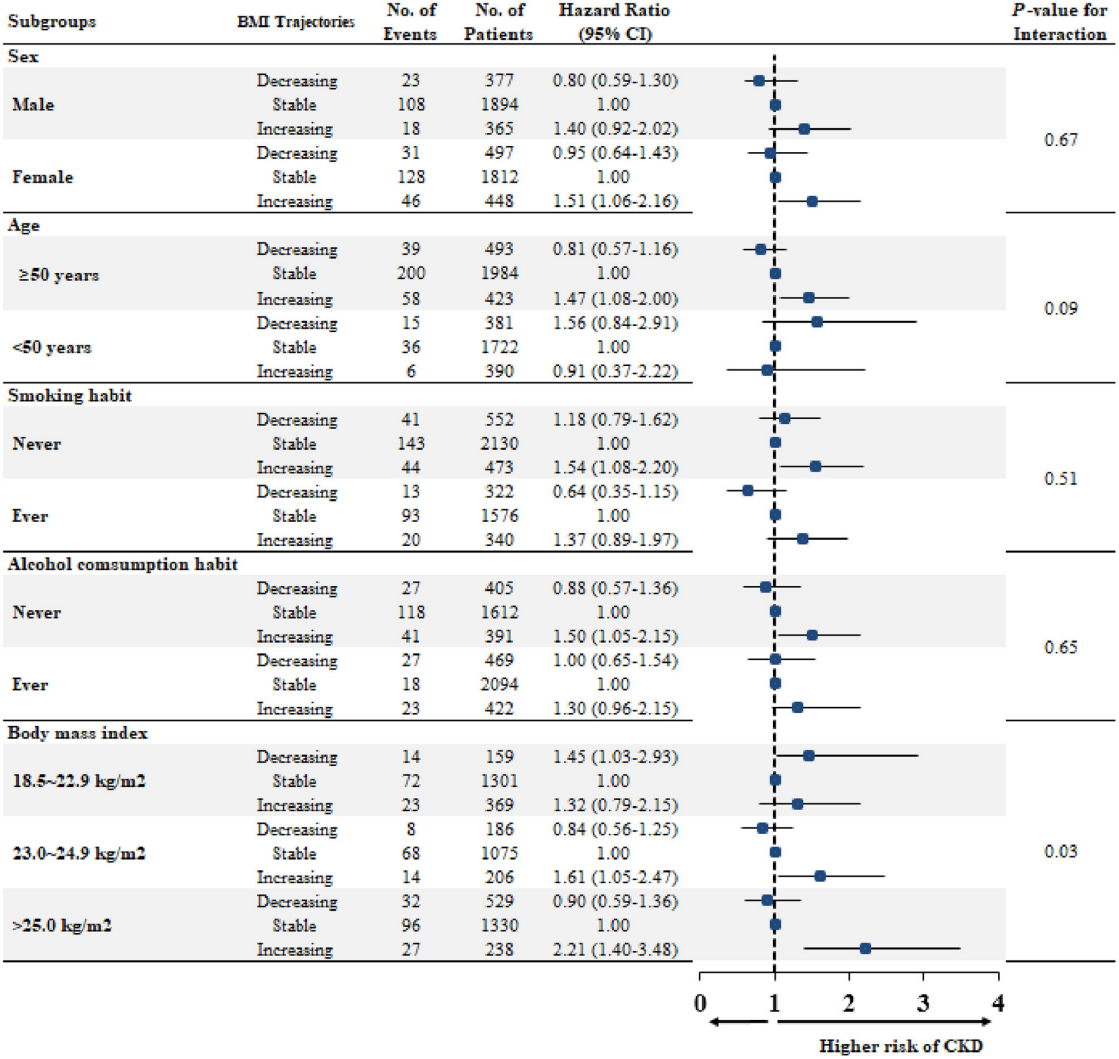
Multivariable-adjusted hazard ratios for CKD developments according to trajectories, stratified by subgroups. Each HR and 95% CI were expressed after adjustment of covariates, including age, sex, the area of residence, smoking, alcohol consumption habit, income, education levels, body mass index, mean arterial pressure, hemoglobin, albumin, fasting glucose, estimated glomerular filtration rate, high-density lipoprotein-cholesterol, triglyceride, and high-sensitive C-reactive protein. BMI, body mass index; No, number; CKD, chronic kidney disease; HR, hazard ratio; CI, confidence interval.

We also tested the effects of weight change on incident CKD in prespecified groups. An increased risk of CKD development associated with an increase in BMI was observed in most subgroups (Figure 3). Notably, this association was particularly evident in the overweight (HR, 1.61; 95% CI, 1.05–2.47) and obese (HR, 2.21; 95% CI, 1.40–3.48) participants, and the harmful association of weight gain was not observed in the normal weight group.

### Changes in Metabolic Profile Parameters

**Figure 4:**
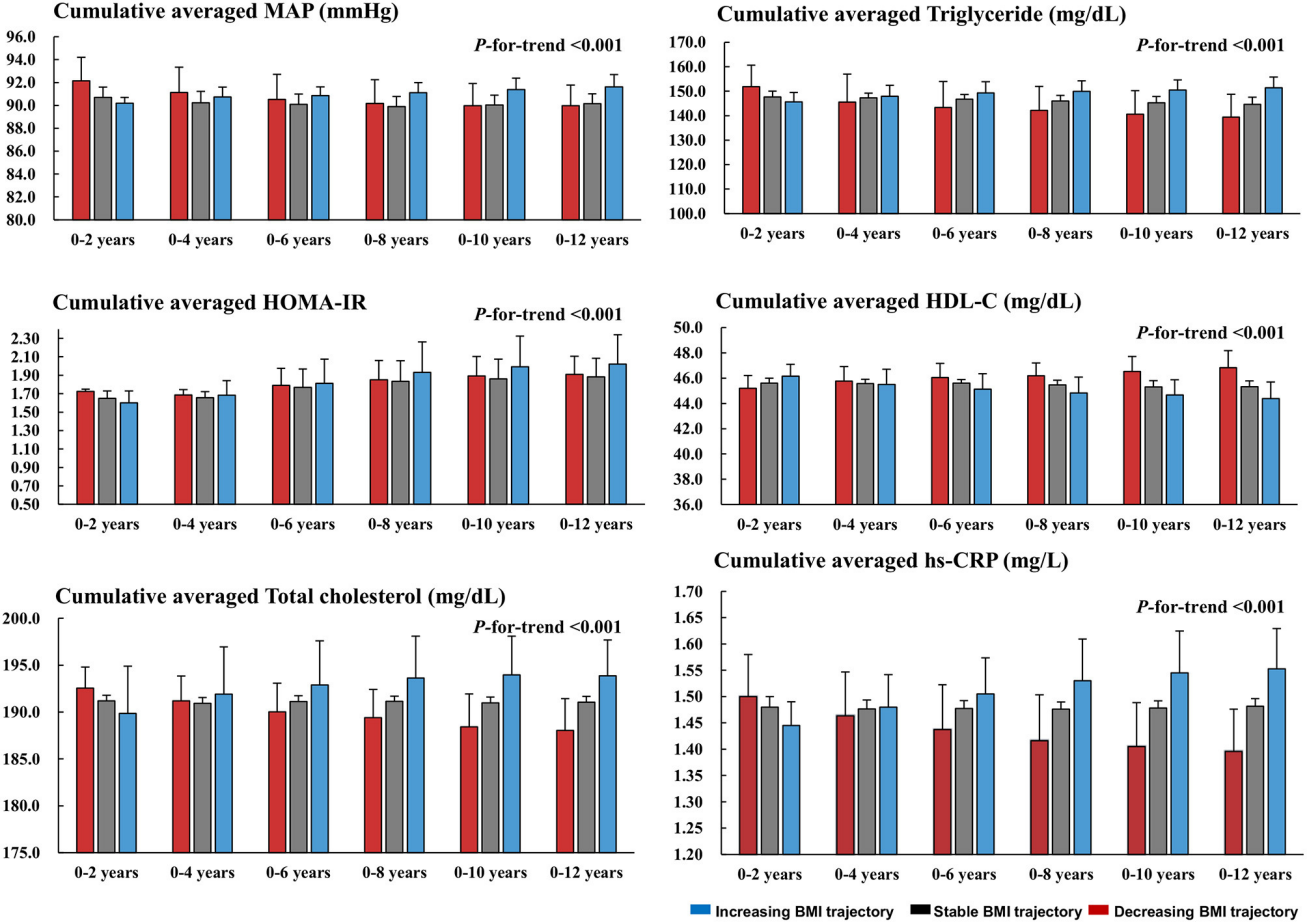
Longitudinal changes in the MAP, HOMA-IR, and metabolic profiles across the trajectories. Cumulative averaged values of MAP, HOM-IR, lipid profiles, and hs-CRP were calculated as the mean of measurements of each parameter during the follow-up period. MAP, mean arterial pressure; HOMA-IR, homeostasis model assessment for insulin resistance; HDL-C, high-density lipoprotein-cholesterol; hs-CRP, high-sensitive C-reactive protein.

We further examined longitudinal changes in BP, insulin resistance, lipid profiles, and an inflammatory marker over time. The BMI changes correlated positively with changes in MAP, total cholesterol, and triglyceride levels, and inversely correlated with changes in the HDL-cholesterol level (Supplementary Table 7). These parameters were significantly worsened over time along with increasing BMI (*P*-for-trend <0.001). Moreover, despite the initial better metabolic profiles and lower inflammation in the increasing BMI group up to year 2, these favorable features were notably lost from year 4, and this group had persistently higher levels of MAP, insulin resistance, total cholesterol, triglycerides, and hs-CRP thereafter (Figure 4).

### Changes in Muscle, Fat, and Muscle-to-Fat Ratios

**Figure 5:**
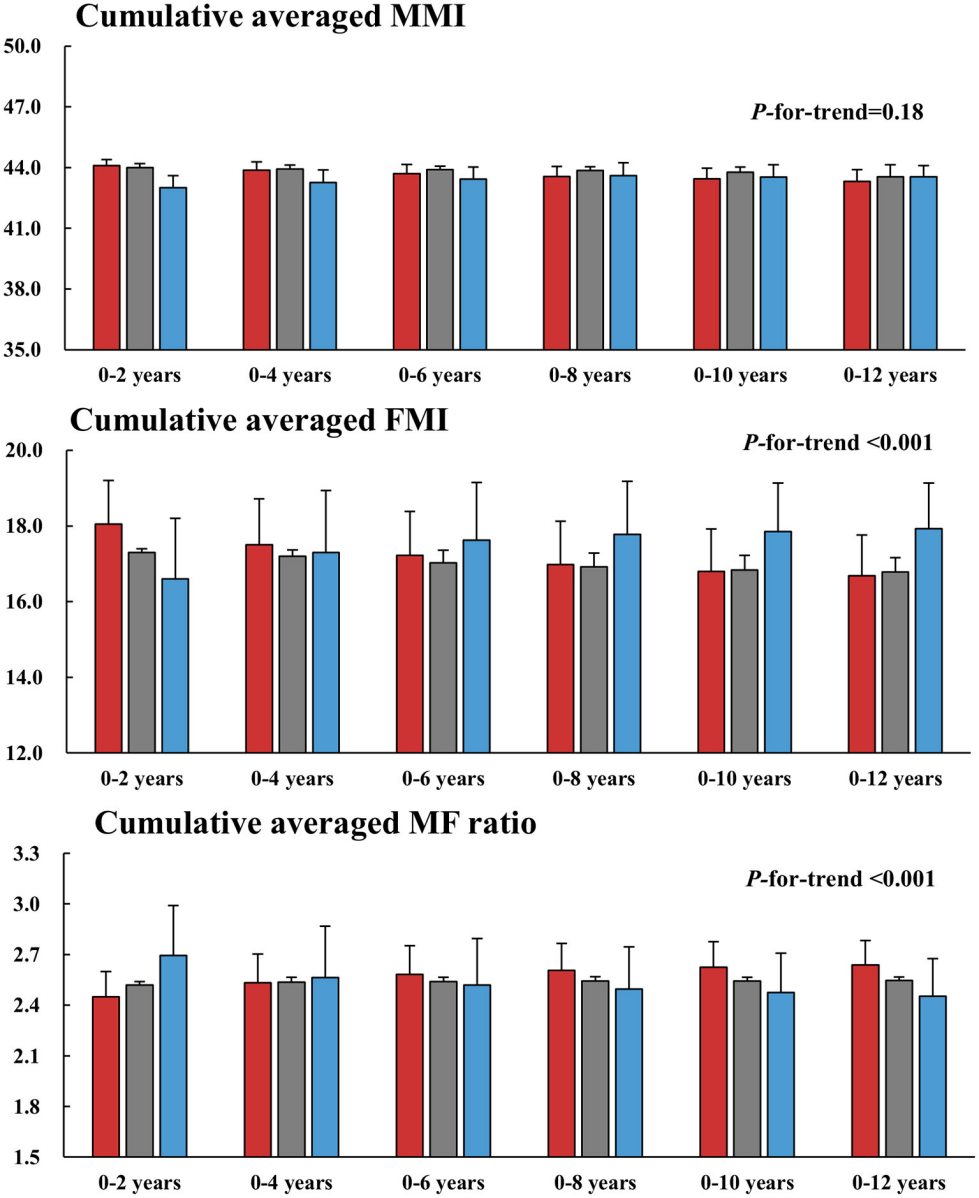
Longitudinal changes in the MMI, FMI, and MF ratio across the trajectories. Cumulative averaged values of the MMI, FMI, and MF ratio were calculated as the mean of measurements of each parameter during the follow-up period. MMI, muscle mass index; FMI, fat mass index; MF, muscle to fat.

Finally, changes in body composition were compared across three trajectories. Muscle mass did not differ among BMI groups throughout the follow-up period. At the baseline, FMI was higher in the decreasing BMI group than in the stable and increasing groups. However, this pattern was reversed from year 2. FMI increased from 15 to 18.2 in the increasing BMI group but decreased from 19.2 to 16.9 in the decreasing group at year 2. FMI remained persistently higher in the increasing BMI group thereafter. Accordingly, the direction of the changes in FMI was the opposite to that of the MMI/FMI ratio (Supplementary Table 8). These changes over time are presented using cumulative averaged means of the MMI, FMI, and MMI/FMI ratio in Figure 5.

## Discussion

The present study showed that weight gain was significantly associated with a higher risk of incident CKD in healthy individuals without comorbidities. This association was attributed to an increase in BP and deterioration of metabolic profiles over time, which were also accompanied by an increase in fat mass and a decrease in the MF ratio, following weight gain. Our findings are noteworthy because we provided in-depth analysis with a special interest in the harmful association of weight gain on kidney disease for long-term observation. This study also highlights the importance of lifestyle modifications to prevent CKD development.

Obesity is an established risk factor for cardiovascular events, stroke, diabetes, and cancer, and is a significant predictor of CKD in the general population (27–30). Although obesity is known as a modifiable factor, it is very difficult to maintain body weight at the optimal level after an intended weight loss. Moreover, regaining weight commonly occurs despite the lifestyle modification with diet and exercise, which can lead to uncertain effects of weight reduction on outcomes. Such a weight-regain phenomenon has recently been documented in the Look Action for Health in Diabetes (AHEAD) study. In this trial, with intensive lifestyle intervention, cardiovascular events, and death in overweight and obese adults with type 2 diabetes did not improve following weight loss (31). However, before the occurrence of weight regain that was obviously observed after 1 year, modest weight loss was associated with significant improvements in BP, glycemic status, and lipid profile, suggesting the necessity for long-term sustained weight loss to see the benefits (32). In this regard, our trajectory analysis has some merits because this method well-disclosed the trends in BMI changes over time, which allowed us to explore the long-term association of weight changes with incident CKD.

The mechanisms underlying the interaction between weight gain and CKD development remain largely presumptive. Ryu et al. postulated that what the subjects experienced may have been caused by worsening cardiovascular or metabolic risk and that they, therefore, had an increased risk of CKD (33). However, these studies did not show concomitant changes in the metabolic profiles of the subjects. In this study, we clearly showed the concomitant worsening of BP, insulin resistance, inflammation, and lipid profiles along with weight gain. It can be presumed that such metabolic derangements adversely affect kidney function. Despite concerns about the inability of BMI to distinguish fat and muscle mass, BMI is highly correlated with adiposity (34), and both increasing weight and body fat carry metabolic risk (35, 36). In our study with body composition analysis, increased BMI was accompanied by decreased MMI and increased FMI, resulting in decreased MF ratios. These changes in body composition toward unhealthy conditions can cause chronic inflammation and negative metabolic effects (37, 38) and lead to structural damage to the kidney and decline in kidney function (39). Thus, our long-term observation on the changes in metabolic profiles, inflammation, and body fat mass can support the link between obesity and CKD.

In contrast to the significantly higher association of weight gain with risk of incident CKD, we did not show that weight loss was not associated with a lower risk of CKD development. Notably, the individuals in the decreasing BMI group were more obese (60.5%) and had unfavorable features of BP, insulin resistance, lipid profiles, and body composition at the baseline compared with the stable and increasing BMI groups. These residual harmful effects of obesity and metabolic abnormalities in the decreasing BMI group might dilute the beneficial effect of weight loss. In addition, the BMI trajectory assessment period was 4 years. Although the unfavorable metabolic parameters were improved during this period, it would take a longer time to exert the clinical benefits of weight loss on CKD development. Nevertheless, when the risk was compared with the increasing BMI group, the decreasing BMI group had a 39% lower risk of incident CKD, suggesting a positive aspect of weight loss against weight gain.

This study has some strengths. To assess the significance of the weight gain itself, we applied strict exclusion criteria; thus, only healthy individuals without comorbidities were included. The use of trajectory analysis is another strength because this method can accurately identify groups with similar trends of changes in BMI over time. Moreover, to avoid the chance of bias due to reversal causality, we analyzed only adverse kidney events that occurred after the BMI trajectory assessment period. Lastly, the KoGES cohort comprises relatively homogenous individuals with a single ethnic group who had undergone extensive assessments for 10 years. This long-term observation period with serial measurements enabled us to comprehensively analyze the associations between CKD development and changes in body weight, metabolic profiles, and body composition.

Several limitations should be also discussed. First, the causal relationship between weight gain and increased risk of CKD development could not fully be established due to the observational study design. Despite the rigorous adjustment, residual confounding is likely to exist in our analyses. Second, BMI has intrinsic problems because it cannot distinguish between muscle and fat, visceral and subcutaneous fat, or peripheral and central adiposity. To minimize this limitation of BMI, we also measured fat and muscle contents by multifrequency BIA and showed that changes in BMI were concomitant to those in muscle and fat mass index. Future studies need more accurate body composition measurements such as dual-energy X-ray absorptiometry to address this issue. Third, the study cohort included asymptomatic middle-aged Korean adults; thus, our findings may not be generalizable to other ethnic groups.

## Conclusion

In conclusion, weight gain was associated with an increased risk of incident chronic kidney disease (CKD) in healthy individuals without comorbid conditions. This relationship was stronger for overweight and obese subjects. An increase in BP and deterioration in metabolic profiles accompanied by increased fat mass may explain the harmful association of weight gain with CKD development. Our findings warn against the potential hazards of weight gain over time on the adverse kidney outcome. More well-designed trials with intensive interventions should address whether weight control can protect against kidney injury.

## Data Availability Statement

The original contributions presented in the study are included in the article/Supplementary Material, further inquiries can be directed to the corresponding author/s.

## Author Contributions

Study concept, design, and acquisition: H-RY and SH. Statistical analysis: H-RY, HK, HWK, YJ, TC, and SH. Analysis and interpretation of data: H-RY, KN, HWK, and SH. Draft manuscript: H-RY, HWK, YJ, HK and SH. Revising it critically for important intellectual content: JP, EK, T-HY, S-WK, and SH. All authors have taken care to ensure the integrity of this work, and the final manuscript has been seen and approved by all authors.

## Funding

This study was supported by the Ministry for Health and Welfare, Republic of Korea [4845-301 and 4851-302], and also supported by a grant of the Korea Health Technology R&D Project through the Korea Health Industry Development Institute (Grant Number: HC15C1129). The funders had no role in study design, data collection, and analysis, decision to publish, or preparation of the manuscript.

## Conflict of Interest

The authors declare that the research was conducted in the absence of any commercial or financial relationships that could be construed as a potential conflict of interest.

## Publisher's Note

All claims expressed in this article are solely those of the authors and do not necessarily represent those of their affiliated organizations, or those of the publisher, the editors and the reviewers. Any product that may be evaluated in this article, or claim that may be made by its manufacturer, is not guaranteed or endorsed by the publisher.
