# Supplementary material for: Increased Risk of Chronic Kidney Disease Associated With Weight Gain in Healthy Adults: Insight From Metabolic Profiles and Body Composition
Source: Front Med (Lausanne). 2021 Sep 28;8:705881. doi: 10.3389/fmed.2021.705881 (PMC8508617; doi:10.3389/fmed.2021.705881)
Supplement: Supplementary file 1 [file Data_Sheet_1.PDF]

*Supplementary Material*

# Increased Risk of Chronic Kidney Disease Associated with Weight Gain in Healthy Adults: Insight from Metabolic Profiles and Body Composition

**Hae-Ryong Yun[1,2], Hyung Woo Kim[2], Tae Ik Chang[3], Ea Wha Kang[1], Young Su Joo[1], Ki Heon Nam[2], HyoungNae Kim[4], Jung Tak Park[2], Tae-Hyun Yoo[2], Shin-Wook Kang[2,5], and Seung Hyeok Han[2*]**

[1]Division of Nephrology, Department of Internal Medicine, Yongin Severance Hospital, Yonsei University College of Medicine, Seoul, Korea

[2]Department of Internal Medicine, College of Medicine, Institute of Kidney Disease Research, Yonsei University, Seoul, Korea

[3]Department of Internal Medicine, National Health Insurance Service Medical Center, Ilsan Hospital, Goyang, Gyeonggi-do, Korea

[4]Division of Nephrology, Soonchunhyang University Hospital, Seoul, Korea

[5]Department of Internal Medicine, College of Medicine, Severance Biomedical Science Institute, Brain Korea 21 PLUS, Yonsei University, Seoul, Korea

***Correspondence:**
Seung Hyeok Han
hansh@yuhs.ac

**Supplementary Table 1. Estimation process for the trajectory groups of determined by the 4-year body mass index measurements.**

| No. of Groups | Functional form | BIC | Average probability | Proportions of groups (%) | | | | |
|---|---|---|---|---|---|---|---|---|
| | | | | Group1 | Group2 | Group3 | Group4 | Group5 |
| 3 | 111 | -18562 | 90.7 | 17.0 | 68.3 | 14.6 | | |
| 3 | 211 | -18492 | 90.8 | 17.4 | 67.9 | 14.6 | | |
| 3 | 121 | -18561 | 90.8 | 17.8 | 68.2 | 13.8 | | |
| 3 | 112 | -18443 | 90.8 | 16.5 | 67.5 | 15.8 | | |
| 3 | 222 | -18373 | 90.9 | 17.6 | 67.2 | 15.2 | | |
| **3** | **212** | **-18352** | **91.0** | **17.0** | **67.2** | **15.8** | | |
| 3 | 202 | -18369 | 90.9 | 18.0 | 67.1 | 14.9 | | |
| 4 | 2222 | -17798 | 88.3 | 3.7 | 52.9 | 34.7 | 8.5 | |
| 4 | 1222 | -17825 | 88.5 | 3.0 | 55.0 | 32.7 | 9.1 | |
| 4 | 2122 | -17836 | 88.5 | 2.8 | 56.0 | 30.8 | 10.2 | |
| 4 | 2212 | -17843 | 87.5 | 4.4 | 50.9 | 36.7 | 7.9 | |
| 4 | 2221 | -17892 | 88.1 | 4.3 | 50.7 | 38.0 | 6.9 | |
| 4 | 1122 | -17864 | 87.8 | 53.7 | 33.2 | 3.6 | 9.4 | |
| 4 | 2112 | -17864 | 87.8 | 3.7 | 53.7 | 33.1 | 9.4 | |
| 4 | 2211 | -17935 | 87.2 | 5.5 | 46.8 | 41.6 | 5.9 | |
| 4 | 1112 | -17911 | 88.0 | 3.2 | 54.7 | 32.4 | 9.6 | |
| 4 | 2111 | -17983 | 87.8 | 4.0 | 52.7 | 35.2 | 7.9 | |
| 4 | 1111 | -18030 | 88.0 | 3.5 | 53.7 | 34.6 | 8.1 | |
| 5 | 22222 | -17448 | 87.2 | 1.5 | 22.2 | 53.6 | 20.1 | 2.3 |
| 5 | 12222 | -17536 | 85.8 | 23.6 | 52.0 | 19.3 | 1.4 | 3.5 |
| 5 | 21222 | -17536 | 85.8 | 1.4 | 23.6 | 52.0 | 19.3 | 3.5 |
| 5 | 22122 | -17444 | 87.1 | 1.4 | 22.8 | 53.4 | 19.7 | 2.4 |
| 5 | 22212 | -17524 | 86.6 | 2.1 | 21.0 | 52.9 | 21.6 | 2.2 |
| 5 | 22221 | -17502 | 87.7 | 1.6 | 19.4 | 55.0 | 22.0 | 1.6 |

The numbers of functional form are considered as following: flat (0), linear (1), quadratic (2) Cubic functional form of slope was not used in trajectories determined by the first 4-years body mass index measurements due to low BIC and discrimination power. Average probability of groups membership is the calculated measure of posterior predicted probabilities for each individual of being a member of a given trajectory group to assess model's discrimination power. Abbreviation: BIC, Bayesian Information Criterion.

**Supplementary Table 2. Changes in BMI and percent changes of BMI across the trajectories**

| BMI during follow-up period, mean (SD), kg/m² | | | | | | | | | | | | |
|---|---|---|---|---|---|---|---|---|---|---|---|---|
| | **2 yr** | ***P*-value** | **4 yr** | ***P*-value** | **6 yr** | ***P*-value** | **8 yr** | ***P*-value** | **10 yr** | ***P*-value** | **12 yr** | ***P*-value** |
| **Decreasing** | 24.5 (3.2) | | 24.0 (3.1) | | 23.9 (3.1) | | 23.9 (3.2) | | 23.8 (3.1) | | 23.9 (3.2) | |
| **Stable** | 24.1 (2.9) | <0.001 | 24.0 (2.9) | <0.001 | 24.0 (2.9) | <0.001 | 24.0 (2.9) | <0.001 | 24.0 (2.9) | <0.001 | 24.0 (2.9) | <0.001 |
| **Increasing** | 24.9 (3.1) | | 25.4 (3.1) | | 25.3 (3.2) | | 25.5 (3.1) | | 25.4 (3.2) | | 25.4 (3.2) | |
| **Changes of BMI, mean (SD), %** | | | | | | | | | | | | |
| | **0-2 yr** | ***P*-value** | **0-4 yr** | ***P*-value** | **0-6 yr** | ***P*-value** | **0-8 yr** | ***P*-value** | **0-10 yr** | ***P*-value** | **0-12 yr** | ***P*-value** |
| **Decreasing** | -4.9(3.4) | | -7.5(3.4) | | -7.6(4.0) | | -7.4(4.7) | | -7.5(4.2) | | -7.5(3.4) | |
| **Stable** | 0.0(2.7) | <0.001 | -0.1(2.7) | <0.001 | -0.2(2.1) | <0.001 | -0.3(2.4) | <0.001 | -0.2(2.6) | <0.001 | -0.2(2.8) | <0.001 |
| **Increasing** | 5.6(3.6) | | 7.6(3.9) | | 7.5(5.4) | | 7.4(5.5) | | 7.6(5.8) | | 7.7(6.0) | |

*Note*, the percent changes of BMI were defined as the difference between baseline BMI and each visit divided by baseline BMI.
Abbreviations: BMI, body mass index; SD, standard deviation; yr, year.

**Supplementary Table 3. Hazard ratios for the incident chronic kidney disease according to body mass index trajectory groups**

| | | Decreasing BMI | Stable BMI | Increasing BMI |
|---|---|---|---|---|
| **Decreasing BMI** | HR 95%CI | - | 1.13 (0.84-1.54) | 1.65 (1.12-2.42) |
| | P-value | | 0.42 | 0.01 |
| **Stable BMI** | HR 95%CI | 0.89 (0.65-1.20) | - | 1.45 (1.08-1.94) |
| | P-value | 0.42 | | 0.01 |
| **Increasing BMI** | HR 95%CI | 0.61 (0.41-0.89) | 0.69 (0.51-0.92) | - |
| | P-value | 0.01 | 0.01 | |

*Note,* HR and 95% CI were expressed after adjustment of confounding factors including age, sex, the area of residence, smoking, alcohol consumption habit, income, education levels, body mass index, mean arterial pressure, hemoglobin, albumin, fasting glucose, estimated glomerular filtration rate, high density lipoprotein-cholesterol, triglyceride, and high sensitive C-reactive protein.

Abbreviation: BMI, body mass index; HR, hazard ratio, CI, confidence interval.

**Supplementary Table 4. Hazard ratios for the incident chronic kidney disease according to body mass index trajectory groups during 4-8 years outcome assessment period**

| 4-8 years | Events | Person-years | Incidence rate 1000 person-years | Delta BMI (%) | Model 1 | Model 2 | Model 3 | Model 4 |
|---|---|---|---|---|---|---|---|---|
| **Decreasing** | 34 | 6538 | 5.1 | -5.6 (-8.8 to -2.0) | 0.99 (0.68-1.44) | 0.99 (0.70-1.0) | 0.97 (0.69-1.39) | 0.96 (0.65-1.41) |
| **Stable** | 146 | 27 881 | 5.2 | -0.2 (-0.5 to 0.2) | Reference | Reference | Reference | Reference |
| **Increasing** | 47 | 5993 | 7.8 | 5.6 (1.9 to 9.3) | 1.51 (1.08-2.09) | 1.52 (1.06-2.11) | 1.55 (1.10-2.11) | 1.63 (1.15-2.31) |

Model 1: a crude analysis without adjustment

Model 2: an adjusted model including age and sex

Model 3: Model 2 plus the area of residence, smoking habit, alcohol consumption habits, income, education level, body mass index, mean arterial pressure, hemoglobin, fasting glucose, and estimated glomerular filtration rate.

Model 4: Model 2 plus high-density lipoprotein-cholesterol, triglyceride, and high sensitive C-reactive protein.

Abbreviation: CKD, chronic kidney disease; BMI, body mass index; HRs, hazard ratios; CI, confidence interval.

**Supplementary Table 5. Hazard ratios for the incident chronic kidney disease according to body mass index trajectory groups after excluding subjects with isolated microscopic hematuria**

| | Events | Person-years | Incidence rate 1000 person-years | Model 1 | Model 2 | Model 3 | Model 4 |
|---|---|---|---|---|---|---|---|
| **Decreasing** | 49 | 7827 | 6.2 | 1.02 (0.75-1.40) | 0.96 (0.70-1.32) | 0.93 (0.69-1.26) | 0.92 (0.66-1.27) |
| **Stable** | 211 | 34593 | 6.0 | Reference | Reference | Reference | Reference |
| **Increasing** | 55 | 7468 | 7.3 | 1.21 (0.90-1.62) | 1.34 (0.98-1.82) | 1.37 (1.01-1.89) | 1.39 (1.03-1.91) |

Model 1: a crude analysis without adjustment

Model 2: an adjusted model including age and sex

Model 3: Model 2 plus the area of residence, smoking habit, alcohol consumption habits, income, education level, body mass index, mean arterial pressure, hemoglobin, fasting glucose, and estimated glomerular filtration rate.

Model 4: Model 2 plus high-density lipoprotein-cholesterol, triglyceride, and high sensitive C-reactive protein.

Abbreviation: CKD, chronic kidney disease; BMI, body mass index; HRs, hazard ratios; CI, confidence interval.

**Supplementary Table 6. Estimated glomerular filtration rate decline according to body mass index trajectory groups**

| | Slope of eGFR decline (mL/min/1.73m$^2$ per year) | 95% CI | *P* for difference between groups | | |
|---|---|---|---|---|---|
| | | | **Decreasing** | **Stable** | **Increasing** |
| **Decreasing** | -1.15 ± 0.06 | -1.28 to -1.01 | - | 0.04 | 0.03 |
| **Stable** | -1.01 ± 0.03 | -1.09 to -0.97 | | - | 0.01 |
| **Increasing** | -1.28 ± 0.08 | -1.42 to -1.15 | | | - |

*Note:* Slope of eGFR decline (mL/min/1.73m$^2$ per year) was expressed as mean ± standard deviation (interquartile range).
Abbreviation: eGFR, estimated glomerular filtration rate; CI, confidence interval.

**Supplementary Table 7. Longitudinal changes in MAP, HOMA-IR, and metabolic profile according to trajectory groups**

| | | | *P* for difference between groups | | |
|---|---|---|---|---|---|
| | **MAP (mmHg per year)** | **95% CI** | **Decreasing BMI** | **Stable BMI** | **Increasing BMI** |
| **Decreasing BMI** | -0.51 | -0.59 to -0.42 | - | <0.001 | <0.001 |
| **Stable BMI** | 0.15 | 0.09 to 0.20 | | - | <0.001 |
| **Increasing BMI** | 0.57 | 0.47 to 0.67 | | | - |
| | **HOMA-IR (per year)** | **95% CI** | **Decreasing BMI** | **Stable BMI** | **Increasing BMI** |
| **Decreasing BMI** | 0.01 | -0.03 to 0.02 | - | <0.001 | <0.001 |
| **Stable BMI** | 0.04 | 0.02 to 0.05 | | - | <0.001 |
| **Increasing BMI** | 0.15 | 0.13 to 0.16 | | | - |
| | **Total cholesterol (mg/dL per year)** | **95% CI** | **Decreasing BMI** | **Stable BMI** | **Increasing BMI** |
| **Decreasing BMI** | -0.67 | -0.92 to -0.41 | - | <0.001 | <0.001 |
| **Stable BMI** | 0.56 | 0.40 to 0.72 | | - | <0.001 |
| **Increasing BMI** | 1.61 | 1.30 to 1.92 | | | - |
| | **Triglyceride (mg/dL per year)** | **95% CI** | **Decreasing BMI** | **Stable BMI** | **Increasing BMI** |
| **Decreasing BMI** | -5.28 | -6.00 to -4.55 | - | <0.001 | <0.001 |
| **Stable BMI** | 0.20 | -1.35 to 0.75 | | - | <0.001 |
| **Increasing BMI** | 2.38 | 1.50 to 3.26 | | | - |
| | **HDL-C (mg/dL per year)** | **95% CI** | **Decreasing BMI** | **Stable BMI** | **Increasing BMI** |
| **Decreasing BMI** | 0.58 | 0.51 to 0.65 | - | <0.001 | <0.001 |
| **Stable BMI** | 0.15 | 0.11 to 0.20 | | - | <0.001 |
| **Increasing BMI** | -0.20 | -0.28 to -0.11 | | | - |

Generalized linear mixed models were used to compare longitudinal changes in the MAP, HOMA-IR, and metabolic profiles. Abbreviation; MAP, mean arterial pressure; HOMA-IR, homeostasis model assessment for insulin resistance; HDL-C, high-density lipoprotein-cholesterol.

**Supplementary Table 8. Changes in body composition across the trajectories**

| **MMI, mean (SD),** muscle mass/height$^2$ | | | | | | | | | | | | |
|---|---|---|---|---|---|---|---|---|---|---|---|---|
| | **2 yr** | ***P*-value** | **4 yr** | ***P*-value** | **6 yr** | ***P*-value** | **8 yr** | ***P*-value** | **10 yr** | ***P*-value** | **12 yr** | ***P*-value** |
| **Decreasing** | 43.8(8.0) | | 43.4(7.9) | | 43.2(7.9) | | 43.0(7.9) | | 42.9(8.0) | | 42.5(8.0) | |
| **Stable** | 44.2(8.0) | 0.43 | 43.8(8.0) | 0.14 | 43.8(8.1) | 0.15 | 43.7(8.1) | 0.12 | 43.3(8.2) | 0.17 | 43.2(8.1) | 0.07 |
| **Increasing** | 43.6(8.1) | | 43.8(8.1) | | 43.9(8.0) | | 44.1(8.2) | | 43.2(8.2) | | 43.4(8.1) | |
| **FMI, mean (SD),** fat mass/height$^2$ | | | | | | | | | | | | |
| | **2 yr** | ***P*-value** | **4 yr** | ***P*-value** | **6 yr** | ***P*-value** | **8 yr** | ***P*-value** | **10 yr** | ***P*-value** | **12 yr** | ***P*-value** |
| **Decreasing** | 16.9(5.7) | | 16.4(5.5) | | 16.4(5.6) | | 16.0(5.7) | | 15.9(5.6) | | 16.0(5.9) | |
| **Stable** | 17.4(5.0) | <0.001 | 17.0(5.0) | <0.001 | 16.5(5.1) | <0.001 | 16.5(5.0) | <0.001 | 16.4(5.1) | <0.001 | 16.5(5.4) | <0.001 |
| **Increasing** | 18.2(5.4) | | 18.7(5.5) | | 18.6(5.9) | | 18.4(5.5) | | 18.2(5.7) | | 18.4(6.0) | |
| **Muscle-to-Fat ratio,** MMI/FMI | | | | | | | | | | | | |
| | **2 yr** | ***P*-value** | **4 yr** | ***P*-value** | **6 yr** | ***P*-value** | **8 yr** | ***P*-value** | **10 yr** | ***P*-value** | **12 yr** | ***P*-value** |
| **Decreasing** | 2.61(1.3) | | 2.70(1.4) | | 2.71(1.4) | | 2.70(1.3) | | 2.72(1.3) | | 2.72(1.2) | |
| **Stable** | 2.54(1.3) | <0.001 | 2.57(1.3) | <0.001 | 2.55(1.3) | <0.001 | 2.56(1.3) | <0.001 | 2.54(1.4) | <0.001 | 2.56(1.1) | <0.001 |
| **Increasing** | 2.40(1.2) | | 2.31(1.1) | | 2.33(1.5) | | 2.38(1.3) | | 2.37(1.2) | | 2.32(1.1) | |

Abbreviations: MMI, muscle mass index; FMI, fat mass index; MF, muscle-to-fat; SD, standard deviation; yr, year.

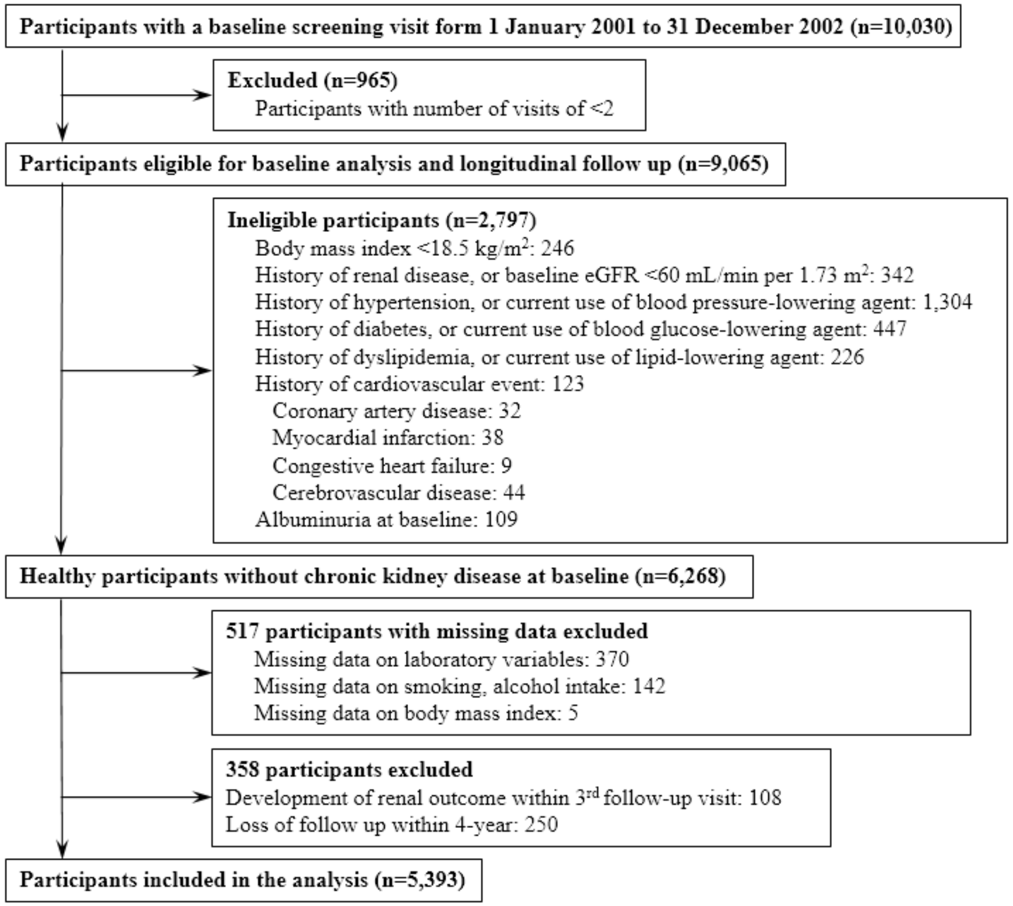


**Supplementary Figure 1. Flow diagram of the study subjects**
Abbreviations: eGFR, estimated glomerular filtration rate (mL/min/1.73 $m^2$).

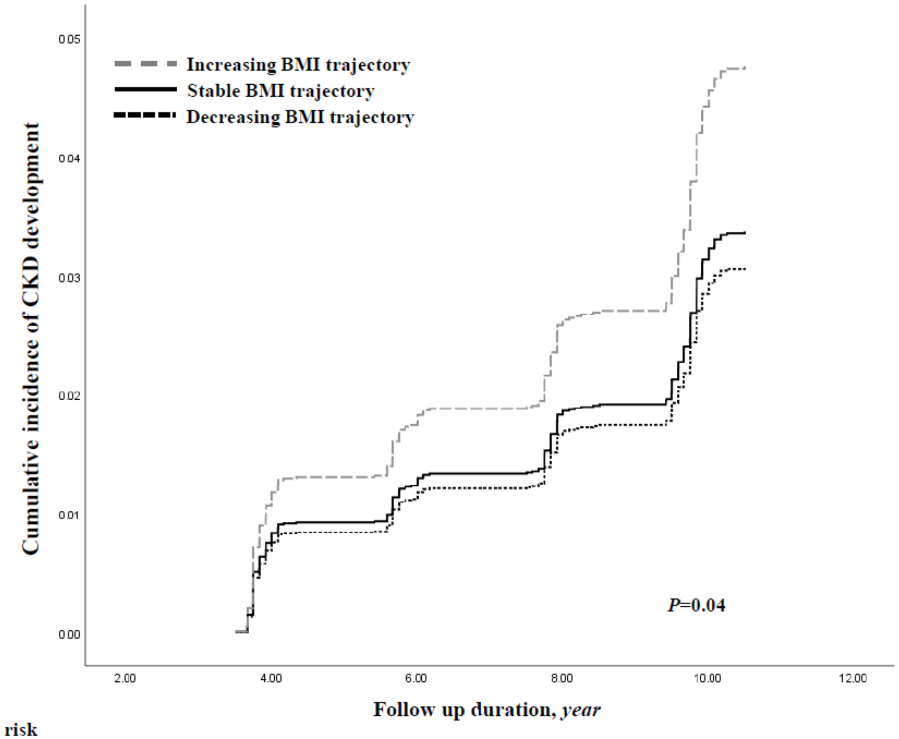


| Number at risk | | | | | | |
|---|---|---|---|---|---|---|
| Increasing BMI | 813 | 812 | 754 | 705 | 596 | 517 |
| Stable BMI | 3706 | 3453 | 3259 | 3045 | 2626 | 2528 |
| Decreasing BMI | 874 | 740 | 679 | 641 | 625 | 594 |

**Supplementary Figure 2. Adjusted cumulative incidence for CKD development according to the BMI trajectories**
Adjusted for age, sex, the area of residence, smoking, alcohol consumption habit, income, education levels, mean arterial pressure, hemoglobin, albumin, fasting glucose, estimated glomerular filtration rate, high-density lipoprotein-cholesterol, triglyceride, and high-sensitivity C-reactive protein.
